# Osteoarticular Involvement-Associated Biomarkers and Pathways in Psoriasis: The Shared Pathway With Ankylosing Spondylitis

**DOI:** 10.3389/fimmu.2022.836533

**Published:** 2022-03-17

**Authors:** Yu-Ping Zhang, Xing Wang, Li-Gang Jie, Yuan Qu, Xiao-Tong Zhu, Jing Wu, Qing-Hong Yu

**Affiliations:** Department of Rheumatology and Clinical Immunology, Zhujiang Hospital, Southern Medical University, Guangzhou, China

**Keywords:** psoriasis, psoriatic arthritis, ankylosing spondylitis, WGCNA, differential gene analysis

## Abstract

Psoriatic arthritis (PsA) is a unique immune-mediated disease with cutaneous and osteoarticular involvement. However, only a few studies have explored the susceptibility of osteoarticular involvement in psoriasis (Ps) at the genetic level. This study investigated the biomarkers associated with osteoarticular participation and potential shared molecular mechanisms for PsA and ankylosing spondylitis (AS).

**Methods:**

The RNA-seq data of Ps, PsA, and AS in the Gene Expression Omnibus (GEO) database were obtained. First, we used the limma package and the weighted gene co-expression network analysis (WGCNA) to identify the potential genes related to PsA and AS. Then, the shared genes in PsA and AS were performed using the GO, KEGG, and GSEA analyses. We also used machine learning to screen hub genes. The results were validated using external datasets and native cohorts. Finally, we used the CIBERSORT algorithm to estimate the correlation between hub genes and the abundance of immune cells in tissues.

**Results:**

An overlap was observed between the PsA and AS-related modules as 9 genes. For differentially expressed genes in AS and PsA, only one overlapping gene was found (COX7B). Gene enrichment analysis showed that the above 9 genes might be related to the mRNA surveillance pathway. The GSEA analyses showed that COX7B was involved in adaptive immune response, cell activation, etc. The PUM1 and ZFP91, identified from the support vector machine, had preferable values as diagnostic markers for osteoarticular involvement in Ps and AS (AUC > 0.7). Finally, CIBERSORT results showed PUM1 and ZFP91 involvement in changes of the immune microenvironment.

**Conclusion:**

For the first time, this study showed that the osteoarticular involvement in psoriasis and AS could be mediated by the mRNA surveillance pathway-mediated abnormal immunologic process. The biological processes may represent the cross talk between PsA and AS. Therefore, PUM1 and ZFP91 could be used as potential biomarkers or therapeutic targets for AS and Ps patients.

## Introduction

Psoriasis (Ps) is a chronic inflammatory skin disease with an incidence rate of 1% to 3%. Arthritis occurring in 10%–40% of psoriasis patients is called psoriatic arthritis (PsA) (1). In most patients with PsA, skin manifestations appear first, preceding arthritis over several years (2). Terminal stages of PsA are generally characterized by joint deformity and/or spinal ankylosis. Osteoarticular involvement represents any symptoms and signs of osteoarticular system, including spondylitis, enthesitis, peripheral arthritis, and dactylitis which affects the patient’s overall quality of life. Previous studies have compared the differences in the genetic background between psoriasis and PsA (3–5). However, as genomically similar but phenotypically distinct diseases, more insight is required at the transcriptomic level to understand the biomarkers or biological pathways associated with the development of osteoarticular involvement in Ps and AS (6).

PsA is a member of the spondyloarthropathy (SpA) family. The SpA diseases, including psoriatic arthritis, ankylosing spondylitis (AS), arthritis associated with inflammatory bowel disease (IBD), reactive arthritis, and undifferentiated spondyloarthropathy, share common genetic backgrounds and present overlapping clinical signs. The ankylosing spondylitis is the prototype of the SpA group. In addition, the bioinformatics background of AS can be a potential representative of osteoarticular involvement, especially axial involvement in spondyloarthropathy (7).

Therefore, this study was carried out to explore osteoarticular involvement-associated biomarkers and pathways in psoriasis patients. We used the limma package and the weighted gene co-expression network analysis (WGCNA) to identify the potential common genes related to PsA and AS. Additionally, we analyzed the published gene expression data at the Gene Expression Omnibus (GEO). We showed that the osteoarticular involvement in psoriasis and AS could be associated with mRNA surveillance pathway-mediated abnormal processes. Moreover, the identified PUM1 and ZFP91 genes were preferable as diagnostic markers for osteoarticular involvement in Ps and AS. To the best of our knowledge, this is the first study to use a systemic bioinformatic analysis approach to explore the gene signatures of osteoarticular involvement in Ps and AS.

## Materials and Methods

### Datasets and Data Preprocessing

We obtained the GEO database’s original gene expression profile data and clinical information (8). We used the keywords “psoriatic arthritis” and “psoriasis” or “ankylosing spondylitis” to search RNA-seq profiles in the GEO database. The following filter criteria were used: the organization used for sequencing should be peripheral blood mononuclear cells (PBMC), and the number of samples of each group should not be less than 10 to ensure the accuracy of the WGCNA. Finally, the GEO datasets numbered GSE61281, GSE25101, and GSE73754 were obtained.

The GSE61281 dataset was used on the GPL6480 platform. The dataset contained 40 samples, including peripheral blood samples from cutaneous psoriasis without inflammatory arthritis (n = 20) and 20 peripheral blood samples from PsA (n = 20). The GSE25101 dataset was used on the GLP6947 platform. This dataset contained 32 samples, including 16 peripheral blood samples from AS patients and 16 peripheral blood samples from healthy controls. Besides, the GSE73754 dataset based on GPL10558 was regarded as an external validation set from the GEO database with 51 cases of AS patients as the experimental group and 20 cases of normal samples as the control group. Moreover, to estimate the diagnostic efficiency of skin lesions other than blood biomarkers, the GSE13355 dataset based on GPL570 was downloaded, including 58 psoriatic lesional samples and 64 non-psoriasis skin samples. The detailed clinical characteristics are shown in **Supplementary Files 1**.

### Screening and Validation of Hub Biomarkers

Firstly, we utilized the limma R package to screen the differential genes (DEGs) from GSE61281 and GSE25101 datasets (9). The screening conditions for the DEGs were the absolute value of |log2 fold change FC|<0.5, and adj. p-value <0.05 was considered as the standard. Next, WGCNA was performed on the obtained DEGs from two datasets. Based on the scale-free topology criterion, the soft-power parameters ranging from 1 to 20 using the “pickSoftThreshold” (package WGCNA) function were screened out. The extracted values were chosen to build an adjacency matrix (10). The most appropriate β value was selected to convert the matrix of correlations to the adjacency matrix and then into a topological overlap matrix. Next, we used the average-linkage hierarchical clustering method to cluster genes based on TOM, where the minimum module size was set at 50. After that, modules with similarities were merged. Finally, Pearson correlation analysis was performed to assess the correlation of the integrated modules with the osteoarticular involvement in Ps and AS.

The support vector machine-recursive feature elimination (SVM-RFE) (11) is a sequential backward feature elimination method based on SVM, which is used to find the optimal hub gene by deleting feature vectors dependent on the e1071 and msvmRFE package (12)) for SVM modeling. We screened core biomarkers using SVM analysis in the above DEGs and intersection of WCGNA. The area under the ROC curve (AUC) was evaluated to assess the diagnostic performance of the core biomarker on the datasets (GSE73754 and GSE13355) and the sequencing data of samples from our hospital.

### Enrichment Analysis

Gene Ontology (GO) category analysis is commonly used for the bioinformatics analysis of large datasets (13). Kyoto Encyclopedia of Genes and Genomes (KEGG) is a database resource for understanding the high-level functions and utilities of the biological system. The results from the GO and KEGG analyses were visualized using the “GOplot” package in R software. Finally, the cluster profile and GSVA packages were used to explore the COX7B gene correlation with specific signaling pathways (14, 15). The gene sets were downloaded from MSigDB (c5.go.bp.v7.4.symbols.gmt) (16). The potential pathways of gene sets and gene expression matrix were detected using gene set enrichment analysis (GSEA).

### Construction of Hub Gene Regulatory Network

The TF–gene interaction pairs with p-values <0.05 were retrieved from TRRUST (17). Finally, the visualization of core gene regulatory network was implemented by using NetworkAnalyst (18).

### Immune Analysis Algorithm

CIBERSORT is a deconvolution algorithm that combines the labeled genomes of different immune cell subpopulations to calculate the proportion of 22 immune cells in tissues (19). Non-parametric correlations (Spearman) were used to determine the correlation between core biomarkers and immune-infiltrated cells.

## Results

### Differential Gene Screening

**Figure 1:**
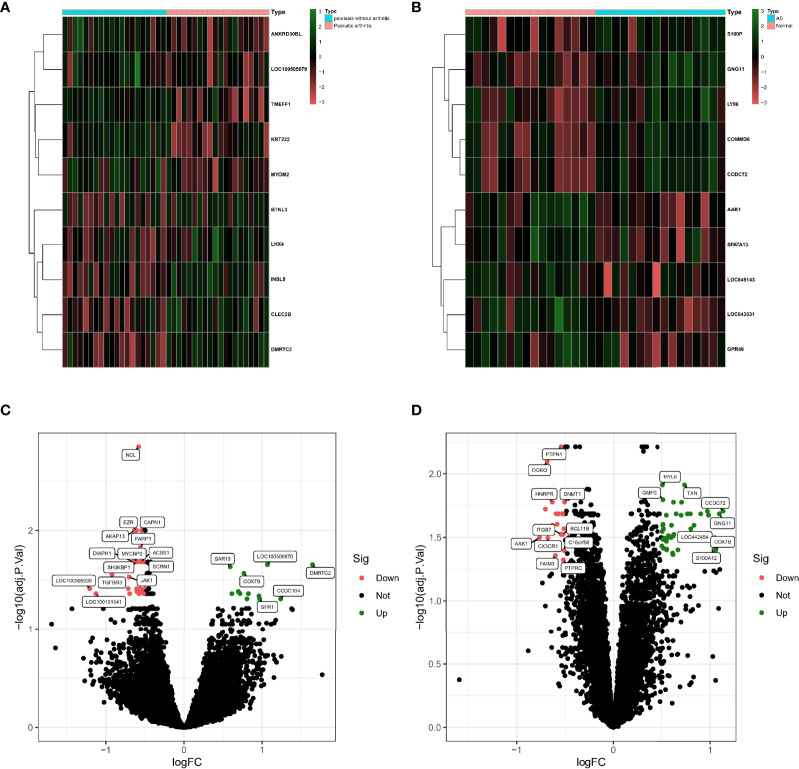
**(A)** Heatmap of DEGs in GSE61281 (n = 37, adj. p < 0.05, |log2 fold change FC| < 0.5). **(B)** Heatmap of DEGs in GSE25101 (n = 62, adj. p < 0.05, |log2 fold change FC| > 0.5). **(C)** Volcano plot of DEGs in GSE61281. **(D)** Volcano plot of DEGs in GSE25101.

Based on the GSE61281 dataset, a total of 37 differential genes (DEGs) were identified. The heatmap demonstrates the top 10 DEGs (**Figure 1A**), obtained using the logFC value. The volcano plot shows the identified DEGs, including 25 upregulated and 12 downregulated (**Figure 1C**). Besides, a total of 62 DEGs were obtained from the GSE25101 dataset, among which 42 genes were upregulated and 20 were found to be downregulated (**Figure 1D**). Heatmaps of the top 10 upregulated and downregulated DEGs are shown in **Figure 1B**.

### Weighted Gene Co-Expression Network Analysis

**Figure 2:**
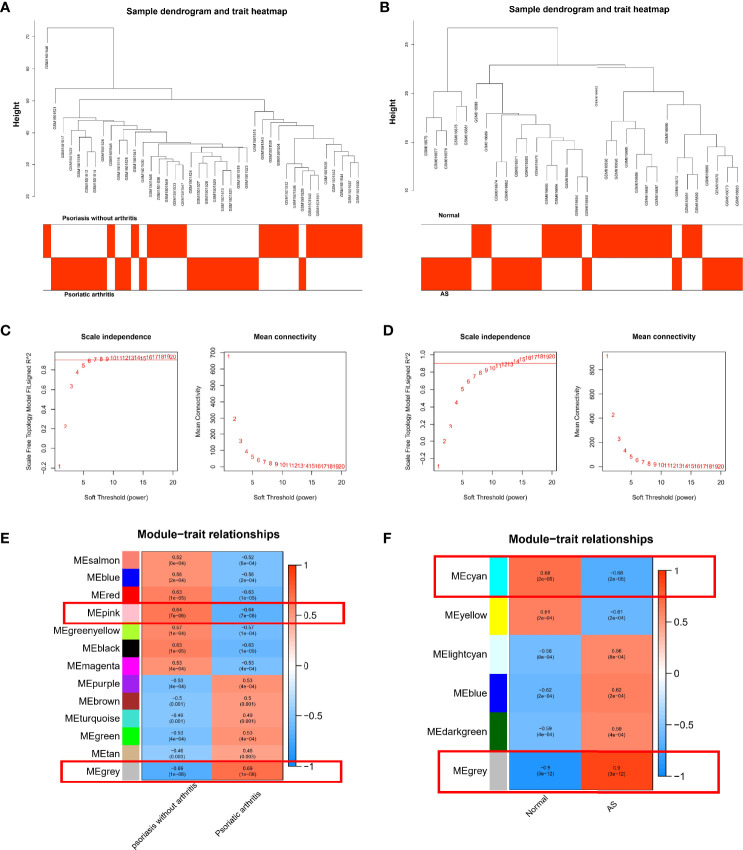
**(A)** Correlation between modules and genes in GSE61281. **(B)** Correlation between modules and genes in GSE25101. **(C)** Determination of soft-thresholding power for GSE61281. **(D)** Determination of soft-thresholding power for GSE25101**. (E)** Heatmap of the correlation between module eigengenes and the occurrence of PsA. **(F)** Heatmap of the correlation between module eigengenes and the occurrence of AS.

We performed WGCNA to investigate the correlation between the clinical information and key genes. The genes with significant differential expression (p < 0.05) were selected as inputs of WGCNA. All samples were clustered in the GSE61281 and GSE25101 datasets, and none of the samples was eliminated (**Figures 2A, B**). In the WGCNA methodology, β = 6 was the optimal soft-power value for GSE61281 (**Figure 2C**), and β = 11 was the optimal soft-power value for GSE25101 (**Figure 2D**). A total of 13 modules were identified in GSE61281, and 6 were identified in GSE25101. Afterward, the correlations between the module and clinical traits were calculated. The gray module had the strongest positive relation with PsA (r = 0.6), while the pink module had the strongest negative relation (r = 0.64) in the GSE61281 database (**Figure 2E**). For AS, the gray module showed the strongest positive correlation (r = 0.9), and cyan had the strongest negative correlation (r = -0.68) in the GSE25101 database (**Figure 2F**).

### Identification of the Shared Genes and TF-mRNA Regulatory Network

**Figure 3:**
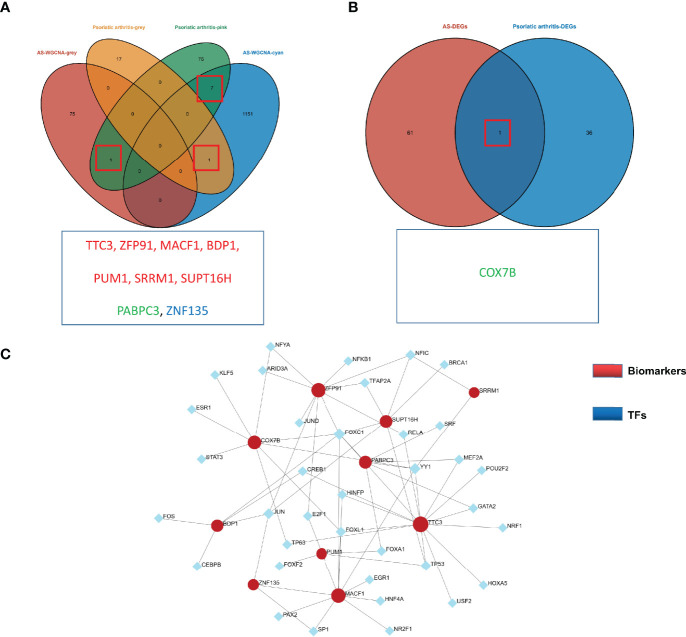
**(A)** Venn diagram shows an overlap of 9 genes in modules between PsA and AS. **(B)** Venn diagram shows an overlap of one DEGs between PsA and AS. **(C)** TF-miRNA regulatory networks. Blue nodes represent transcription factors (TFs), and red nodes represent biomarkers. Black edges represent regulatory relationships between TFs and biomarkers.

An overlap was observed between the PsA and AS modules as a total of 9 genes (TTC3, ZFP91, MACF1, BDP1, PUM1, SRRM1, SUPT16H, PABPC3, ZNF135) (**Figure 3A**). For DEGs, only one overlapping gene was found (COX7B) (**Figure 3B**). These genes might be involved in the pathogenesis of osteoarticular involvement in psoriasis and AS, and have a sharing relationship. Therefore, we searched for an upstream transcriptional regulator that possibly regulated the above 10 genes by the JASPAR database based on the above results. There were 46 nodes and 67 edges found in total (**Figure 3C**).

### Identification of the Shared Pathways

**Figure 4:**
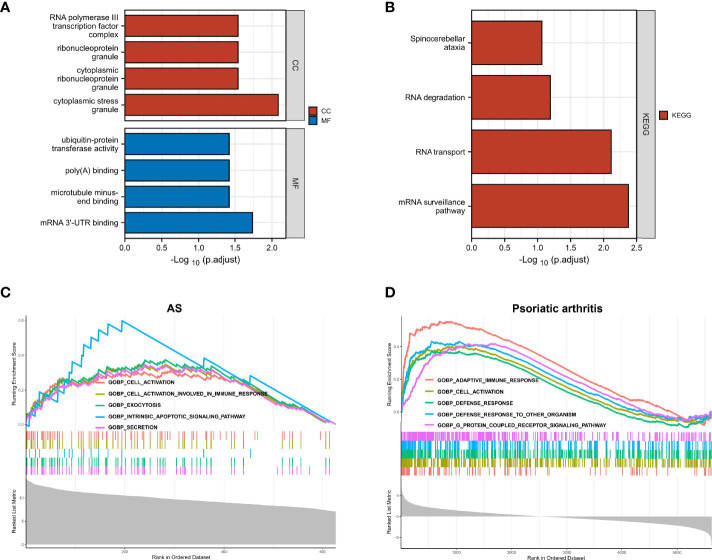
**(A)** GO enrichment analysis results for 9 genes screened by WGCNA. **(B)** KEGG enrichment analysis results for 9 genes screened by WGCNA. **(C)** Single-gene GSEA analyses results for DEGs of AS. **(D)** Single-gene GSEA analyses results for DEGs of PsA.

We further explored the common regulatory pathway that 9 genes were screened by WGCNA and 1 gene was selected as overlapping DEGs. The GO and KEGG enrichment analyses were performed in the above 9 genes. The GO analysis showed that the above 9 genes might be related to the cytoplasmic stress granule, cytoplasmic ribonucleoprotein granule, ribonucleoprotein granule, and RNA polymerase III transcription factor complex (**Figure 4A**). The KEGG analysis showed that these genes might be correlated with the mRNA surveillance pathway (**Figure 4B**). Finally, we performed a single-gene GSEA analysis. The only shared differential gene of AS and PsA samples (COX7B) might participate in several biological processes, including adaptive immune response and cell activation (**Figures 4C, D**). Hence, we made a conjecture that the occurrence of osteoarticular involvement in psoriasis and AS was likely mediated by mRNA surveillance pathway-mediated abnormal immunologic processes.

### Identification of Potential Shared Diagnostic Gene Targets Based on the Machine Learning Algorithm

**Figure 5:**
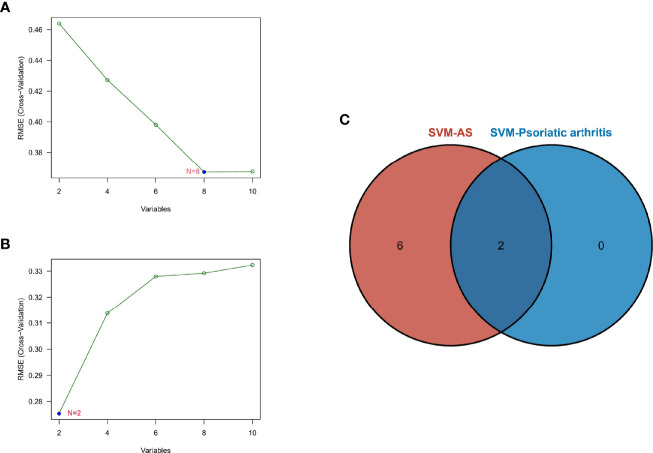
**(A)** SVM-RFE algorithm to screen diagnostic markers in the GSE25101 database. **(B)** SVM-RFE algorithm to screen diagnostic markers in the GSE61281 database. **(C)** Venn diagram shows the optimal diagnostic biomarkers.

SVM-RFE is a machine learning method based on the support vector machines used to find the best core gene by deleting feature vectors produced by SVM. Based on the 10 shared genes, a total of 8 genes were identified as the biomarkers in GSE25101 (**Figure 5A**), and 2 genes in the GSE61281 dataset (**Figure 5B**). These biomarkers might have diagnostic value. Finally, we identified PUM1 and ZFP91 as the optimal diagnostic biomarkers for osteoarticular involvement in psoriasis and AS (**Figure 5C**).

### Validation of Diagnostic Shared Biomarkers

**Figure 6:**
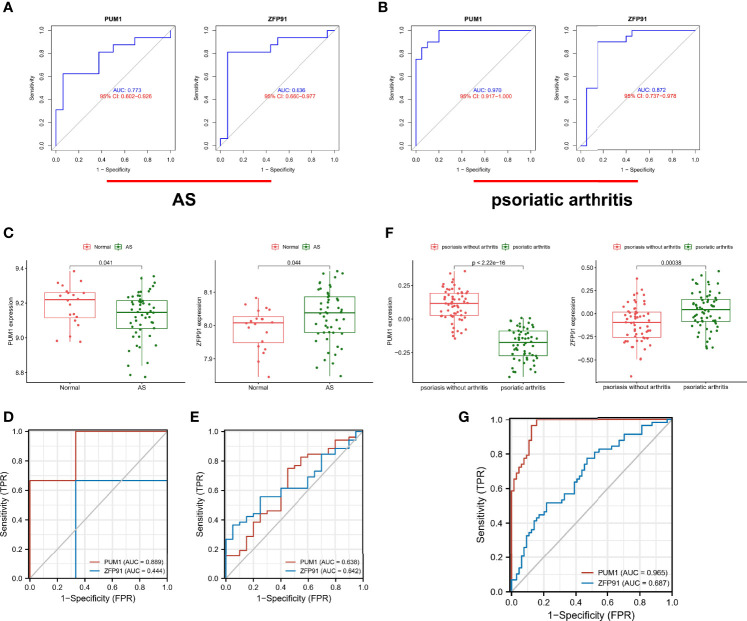
Validation of diagnostic shared biomarkers. **(A)** The ROC curve of the diagnostic efficacy verification in GSE25101. **(B)** The ROC curve of the diagnostic efficacy verification in GSE61281. **(C)** The shared biomarkers in GSE73754 showed significant differences, with p value < 0.05. **(D)** The ROC curve of the diagnostic efficacy verification in data of samples from our hospital. **(E)** The ROC curve of the diagnostic efficacy verification in GSE73754. **(F)** The shared biomarkers in GSE13355 showed significant differences, with p value < 0.05. **(G)** The ROC curve of the diagnostic efficacy verification in GSE13355.

Furthermore, we identified the diagnostic efficacy of the shared biomarkers. In the GSE25101 dataset, these two biomarkers had preferable values as diagnostic markers: PUM1 (AUC = 0.733) and ZFP91 (AUC = 0.836) (**Figure 6A**). The same ROC analysis was performed again for the above biomarkers in the GSE61281 dataset. Each biomarker showed the robust capacity of predictive performance: PUM1 (AUC = 0.970) and ZFP91 (AUC = 0.872) (**Figure 6B**). We then performed external validation for the diagnostic efficacy of PUM1 and ZFP91 in GSE73754, similar to blood RNA-sequencing in AS. The results showed that they show significant differences in expression (**Figure 6C**) and good diagnostic accuracy for detection of AS: PUM1 (AUC = 0.638) and ZFP91 (AUC = 0.642) (**Figure 6E**). In data of samples from our hospital, only PUM1 (AUC = 0.0.889) showed good prediction efficacy, while ZFP91 showed weak prediction efficacy (**Figure 6D**). Meanwhile, we analyzed RNA-seq datasets of skin samples of psoriasis (GSE13355). Both PUM1 and ZFP91 showed significant differences between groups (**Figure 6F**). Each biomarker had potent predictive performance: PUMI (AUC = 0.965) and ZFP91 (AUC = 0.687) (**Figure 6G**).

### Immune Infiltration Analysis of Shared Biomarkers

**Figure 7:**
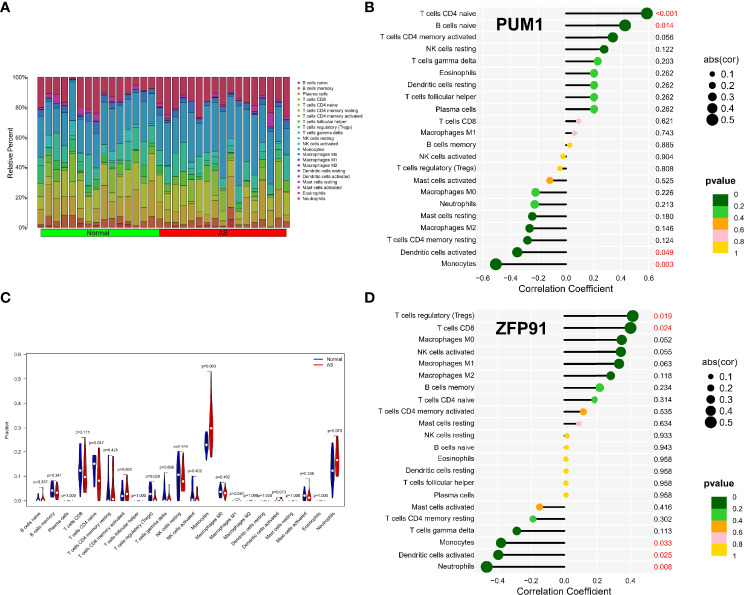
Immune infiltration analysis of shared biomarkers in AS**. (A)** The barplot of immune cell infiltration. **(B)** Correlation between PUM1 and infiltrating immune cells. **(C)** Violin diagram of the proportion of 22 types of immune cells. The red marks represent the difference in infiltration between the two groups of samples. **(D)** Correlation between ZFP91 and infiltrating immune cells.

**Figure 8:**
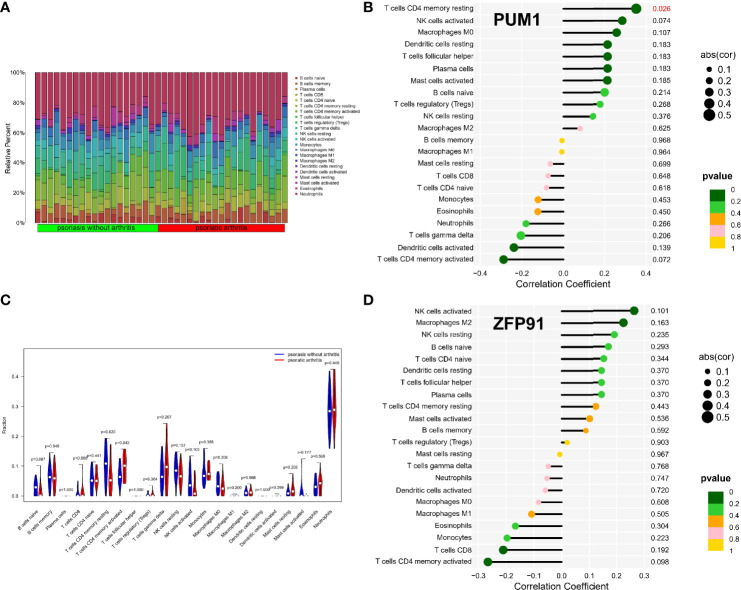
Immune infiltration analysis of shared biomarkers in PsA**. (A)** The barplot of immune cell infiltration. **(B)** Correlation between PUM1 and infiltrating immune cells. **(C)** Violin diagram of the proportion of 22 types of immune cells. The red marks represent the difference in infiltration between the two groups of samples. **(D)** Correlation between ZFP91 and infiltrating immune cells.

The enrichment analysis results showed that immunity plays an important role in developing osteoarticular involvement in psoriasis and AS. The CIBERSORT algorithm was used to analyze the abundances of immune cells in different samples. Bar graphs show the significant differences in the percentage of B cell and macrophage populations between AS and psoriasis samples (**Figures 7A**, **8A**). Compared with the normal sample, CD4-naive T cells and regulatory T cells (Tregs) were decreased in the AS sample, while monocytes were increased (**Figure 7B**). However, compared with psoriasis without arthritis, T cells CD4 memory activated were increased in the psoriasis with osteoarticular involvement, while T cells CD4 memory resting decreased (**Figure 8B**). Moreover, the correlation of the biomarkers and content of different immune cells was explored. In AS samples, PUM1 had a significant positive correlation with naive B cells and CD4-naive T cell content. In contrast, there was a significant negative correlation between PUM1 and both monocytes and activated dendritic cell content (**Figure 7C**). ZFP91 had a significant positive correlation with both CD8 T cells and Tregs and a significant negative correlation with monocytes, activated dendritic cells, and neutrophils (**Figure 7D**). In PsA samples, only PUM1 had a significant positive correlation with memory resting CD4T cells (**Figure 8C**). The statistical analysis showed no significant differences between ZFP91 and other immune cell content (**Figure 8D**).

## Discussion

The characteristics and phenotype of osteoarticular involvement of PsA is consistent with the phenotype of spondyloarthritis (20–22). Meanwhile, ankylosing spondylitis is the prototype of spondyloarthritis, with the typical characteristics of osteoarticular involvement of SpA, including spondylitis, enthesitis, peripheral arthritis, and dactylitis. Osteoarticular involvement of SpA may have a variety of manifestations, but the pathological mechanism seems to be similar, such as pathologic new bone formation, typically occurring at sites of soft tissue surrounding the entheses, and synovitis with more vascularity, a greater infiltration of neutrophils than rheumatic arthritis (22). Therefore, considering the phenotypic disturbances in the PsA group, we studied osteoarticular involvement as a clinical feature that differentiated PsA from Ps. The WGCNA approach was successfully applied in various diseases to identify common risk biomarkers and pathways associated with the different disease phenotypes (23–25). In the present study, the shared gene co-expression module of AS and PsA revealed that the osteoarticular involvement in Ps and AS could be associated with the mRNA surveillance pathway-mediated abnormal immunologic process and identified PUM1 and ZFP91 had preferable value as diagnostic markers for osteoarticular involvement, especially axial involvement in Ps and AS.

The mRNA surveillance pathway is a quality-control physiological mechanism that degrades and detects abnormal mRNAs, including non-stop mRNA decay (NSD), nonsense-mediated mRNA decay (NMD), and no-go decay (NGD) (**Supplementary Figure S1**). The present study results show that the overlapping genes between the PsA and AS modules in WGCNA were correlated with the mRNA surveillance pathway. This observation was suggestive of the pathophysiologic mechanism of osteoarticular involvement in Ps association with the mRNA surveillance pathway. PsA is a chronic immune-mediated rheumatic disease. Studies have reported that the posttranscriptional regulation of gene expression plays a vital role in rheumatic disease (26, 27). However, most studies have focused on microRNA and related pathways, while only a few focused on the mRNA surveillance pathway (28, 29). Notably, no studies have been conducted for assessing the relationship of osteoarticular involvement in Ps and mRNA surveillance pathway; thus, the specific mechanism needs to be further studied and confirmed. Moreover, multiple factors are involved in the immunologic process and mRNA surveillance pathway; the results of this study represent the preliminary exploration.

This pathway was not reported for Ps or PsA, but it was confirmed to be involved in the mechanism of other immune-mediated disease. The abnormal mRNA surveillance machinery causes abnormal activation of immunologic defense programs, resulting in autoimmune diseases (30). As an immune-mediated disease, the process of osteoarticular involvement on psoriasis is probably associated with this mechanism. However, considering the complexity of the mRNA surveillance pathway, the specific mechanism still needs to be confirmed by further studies.

Meanwhile, the COX7B gene is shared between the DEGs of PsA and AS. Single-gene GSEA analyses suggested that it might be associated with several immunologic processes, including adaptive immune response and cell activation. Moreover, the immunologic process and inflammatory response of SpA including PsA are different from other inflammatory arthritis (31). However, concerning immune response and epigenetics, Ps and PsA were usually considered as a group of diseases in most studies; therefore, we barely knew the difference of immunologic processes between psoriasis and PsA (32). The main reason probably lies in the challenge to obtain samples of involved joints. In this case, the analysis of the RNA-seq profiles of PBMCs is an alternative. Our study showed differences in the Ps- and PsA-immune microenvironment, correlating with the biomarkers of osteoarticular involvement.

Immune infiltration analysis revealed the involvement of several specialized immune cell populations, suggesting differences in the immune response between PsA and Ps. Compared with psoriasis without arthritis, T cells CD4 memory activated were increased in psoriasis with osteoarticular involvement, while T cells CD4 memory resting were decreased. Similarly, recent studies have demonstrated that tissue-resident memory CD8+ T cells from the skin helped differentiate psoriatic arthritis from psoriasis (33). Of note, CD4+T cells play an important role in PsA (34).

Furthermore, our studies have shown that PUM1 and ZFP91 might be useful biomarkers or potential therapeutic targets for osteoarticular involvement in Ps and AS due to their involvement in the pathophysiology of osteoarticular involvement of PsA. Previously few studies directly focused on the relationship between these two markers and SpA. In terms of the inflammatory process, ZFP91 plays a role in the non-canonical NF-kB pathway (35) and is required to maintain regulatory T cell homeostasis (36). With respect to the pathological bone formation, the PUM1 gene was differentially expressed in osteoporosis-related cells (37). Therefore, we speculated that PUM1 and ZFP91 might participate in the process of osteoarticular involvement by activated inflammation or pathological bone formation. Further, we found a significant positive correlation between PUM1 and T-cells CD4 memory resting in psoriatic samples. T-cells CD4 memory resting had a negative correlation with osteoarticular involvement in Ps. We speculate that PUM1 may mediate bone and joint involvement in Ps by inhibiting T-cells CD4 memory resting.

Our study had few limitations. Transcriptome analysis of peripheral blood is a useful approach to compare genotype-similar but phenotype-distinct diseases, or even diseases with different-organ involvement. However, the expression profiling of peripheral blood mononuclear cells should be confirmed by the expression profiling of the target organ. Considering the phenotypic disturbances of the PsA group, the current study is the preliminary exploration of genetic factors related to osteoarthritis involvement in Ps, hoping to provide some meaningful directions for follow-up research.

To conclude, this study is the first to explore the pathways and biomarkers of osteoarticular involvement in psoriasis and AS using the bioinformatics tool. Besides, our study revealed the mRNA surveillance pathway and two diagnostic gene biomarkers (PUM1 and ZFP91) for the osteoarticular involvement in psoriasis and AS. In addition, by exploration of the two typical diseases AS and PsA, this study may also provide a new perspective to the pathogenesis of SpA.

## Data Availability Statement

The datasets presented in this study can be found in online repositories. The names of the repository/repositories and accession number(s) can be found in the article/**Supplementary Material**.

## Ethics Statement

The studies involving human participants were reviewed and approved by the Ethics committee of Zhujiang Hospital of Southern Medical University. The patients/participants provided their written informed consent to participate in this study.

## Author Contributions

Y-PZ and XW designed and conducted the whole research. YQ and L-GJ, X-TZ collected the GEO datasets and carried out initial data analysis. Y-PZ, XW, JW, and Q-HY completed the data analysis and drafted the manuscript. JW and Q-HY revised and finalized the manuscript. All authors contributed to the article and approved the submitted version. Y-PZ and XW have contributed equally to this work.

## Funding

This study received the funding of the Guangdong Basic and Applied Basic Research Fund (grant number 2020A1515010039) and the National Natural Science Foundation of China (grant no. 81771727). The National Natural Science Foundation of China (grant no. 82074160).

## Conflict of Interest

The authors declare that the research was conducted in the absence of any commercial or financial relationships that could be construed as a potential conflict of interest.

## Publisher’s Note

All claims expressed in this article are solely those of the authors and do not necessarily represent those of their affiliated organizations, or those of the publisher, the editors and the reviewers. Any product that may be evaluated in this article, or claim that may be made by its manufacturer, is not guaranteed or endorsed by the publisher.
