# Supplementary figures and images for: Osteoarticular Involvement-Associated Biomarkers and Pathways in Psoriasis: The Shared Pathway With Ankylosing Spondylitis
Source: Front Immunol. 2022 Mar 17;13:836533. doi: 10.3389/fimmu.2022.836533 (PMC8969572; doi:10.3389/fimmu.2022.836533)

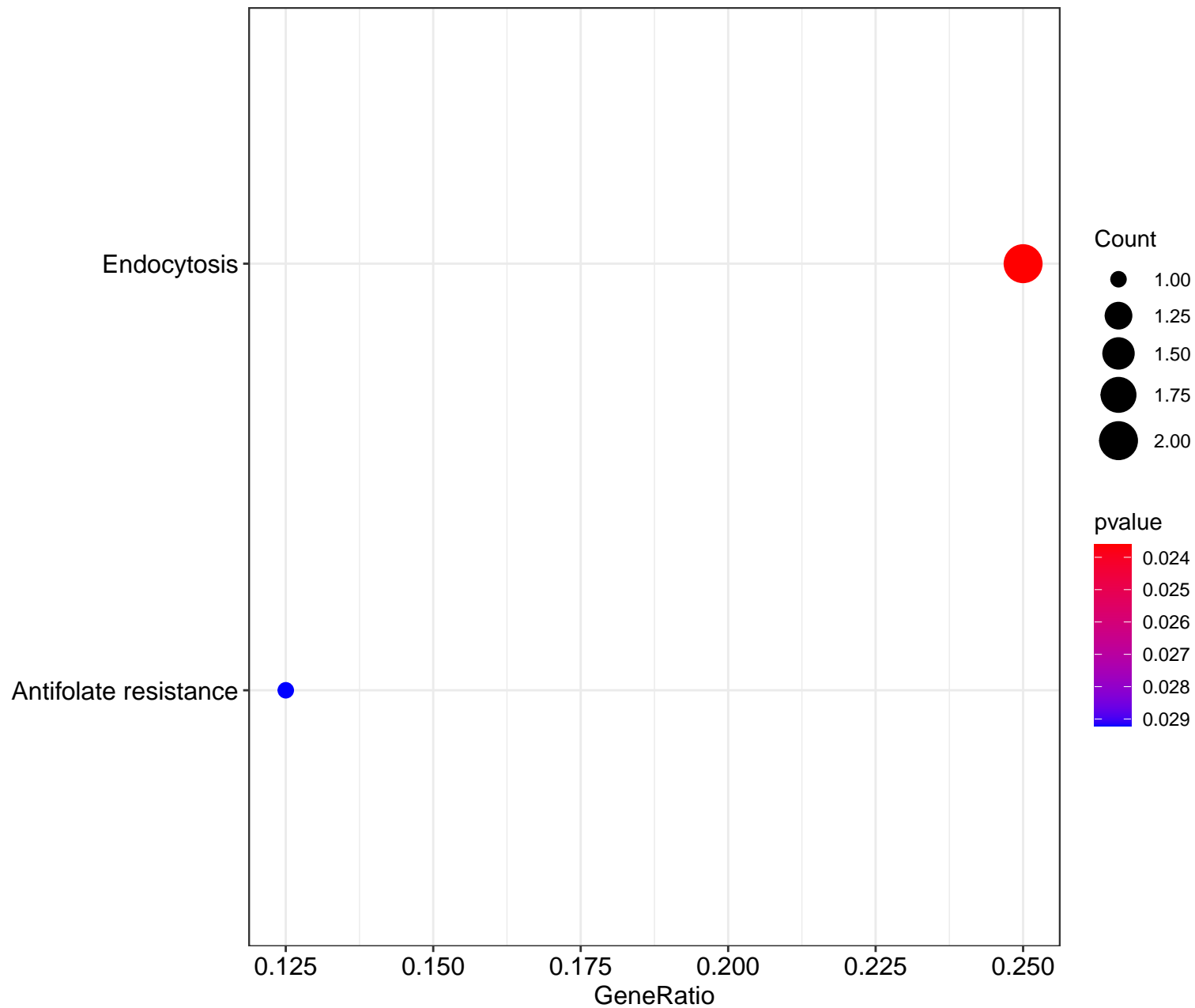

Supplement: Supplementary file 2 [file DataSheet_2.zip › GO and KEGG analysis for grey module/bubble.pdf]

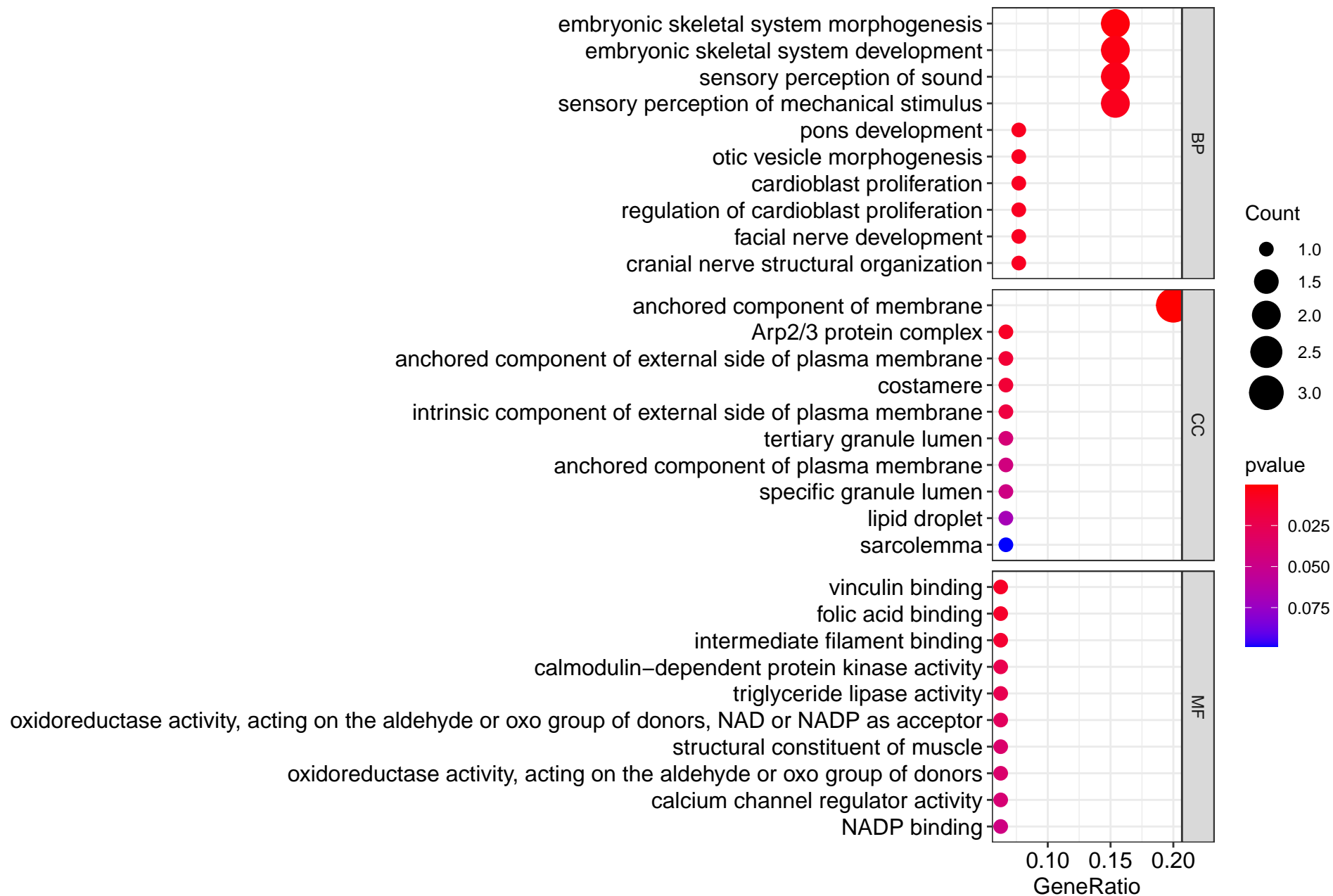

Supplement: Supplementary file 2 [file DataSheet_2.zip › GO and KEGG analysis for grey module/go-bubble.pdf]

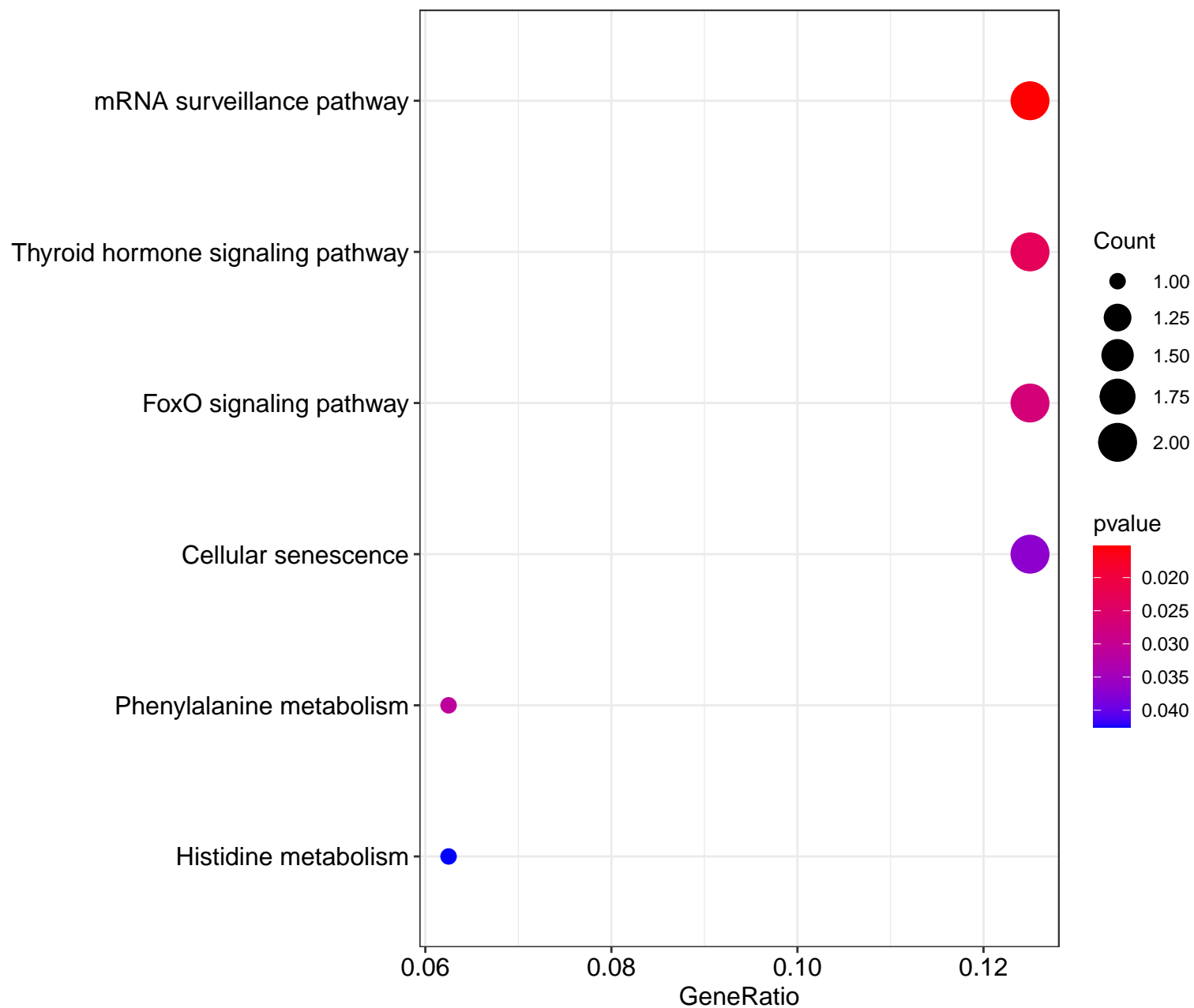

Supplement: Supplementary file 3 [file DataSheet_3.zip › GO and KEGG analysis for pink module/bubble.pdf]

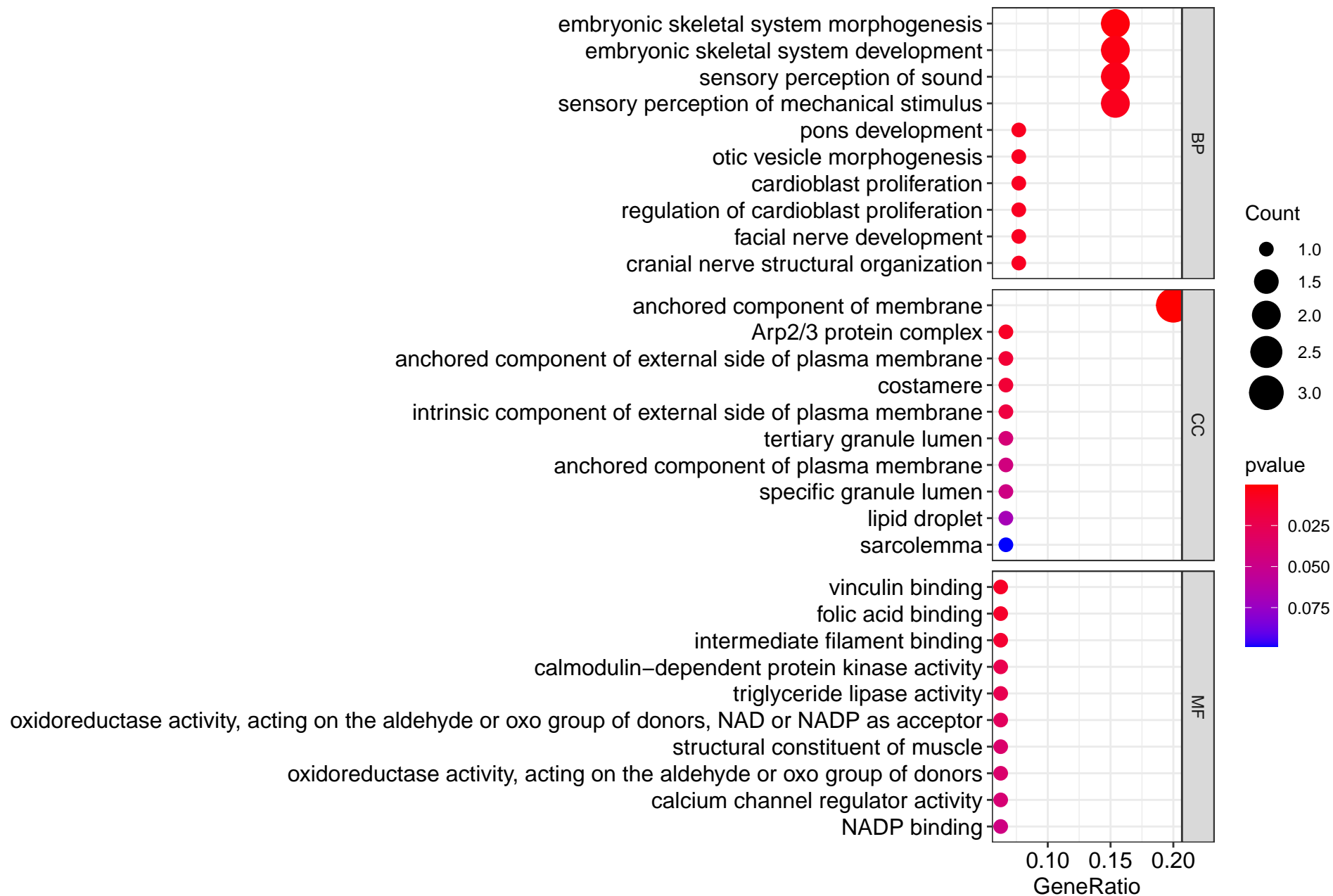

Supplement: Supplementary file 3 [file DataSheet_3.zip › GO and KEGG analysis for pink module/go-bubble.pdf]
